# Real-world performance of faricimab for neovascular age-related macular degeneration and diabetic macular edema: experience from the BrazilRetNet multicenter study. Report 1

**DOI:** 10.1186/s40942-026-00860-z

**Published:** 2026-04-22

**Authors:** Vinicius Kniggendorf, Thiago C. Silveira, Vitor D. M. Costa, Danilo S. Soriano, Caio V. Regatieri, Renata F. Sugai, Arnaldo Bordon, Giovanna S. Nutels, Ana L. Peixoto, Carlos Augusto Moreira-Neto, Joao Victor C. Miranda, Marcio B. Nehemy, Francisco M. Damico, Leandro C. Zacharias, Daniel Lavinsky, Cleide G. Machado, Carlos Eduardo D. Veloso, Francyne V. Cyrino, Wener Cella, Luiz Roisman, Oswaldo F. Brasil, Gabriel C. Andrade, Francisco Stefanini, Fernando K. Malerbi, Fernando M. Penha, BrazilRetNet, Brazilian Retinal Research Network

**Affiliations:** 1 Universidade Federal de Sao Paulo Escola Paulista de Medicina, São Paulo, SP Brazil; 2 Hospital Oftalmologico de Brasilia, Brasília, DF Brazil; 3 Vera Cruz Oftalmologia, Campinas, SP Brazil; 4 Fundacao Roberto Rocha Brito, Campinas, SP Brazil; 5 Hospital das Clínicas da Faculdade de Medicina da Universidade de Sao Paulo, São Paulo, SP Brazil; 6 Centro de Cirurgia Ocular Jardins, São Paulo, SP Brazil; 7 UPO oftalmologia, São Paulo, SP Brazil; 8 Retina and Vitreous, BOS Hospital Oftalmologico de Sorocaba, Sorocaba, SP Brazil; 9 OftalmoFurman, Sorocaba, SP Brazil; 10 Instituto Brasileiro de Oftalmologia (IBOL), Rio de Janeiro, RJ Brazil; 11 Universidade Federal de Minas Gerais, Belo Horizonte, MG Brazil; 12 Hospital de Olhos do Parana, Curitiba, PR Brazil; 13 Universidade Federal do Rio Grande do Sul, Porto Alegre, RS Brazil; 14 Oftalmologia, Universidade de Sao Paulo Faculdade de Medicina, São Paulo, SP Brazil; 15 Núcleo de Oftalmologia Especializada, Belo Horizonte, MG Brazil; 16 Universidade de Sao Paulo Hospital das Clínicas da Faculdade de Medicina de Ribeirao Preto, Ribeirao Preto, SP Brazil; 17 Hospital de Referência Oftalmológica, São Luís, MA Brazil; 18 Orbit Ophthalmo Learning, Rio de Janeiro, RJ Brazil; 19 Faculdade de Ciencias Médicas da Santa Casa de Sao Paulo, São Paulo, SP Brazil; 20 Edge Health, São Paulo, SP Brazil; 21 Universidade Regional de Blumenau, Blumenau, SC Brazil; 22 Botelho Hospital da Visão, Rua 2 de Setembro, Blumenau, 2958 SC Brazil

**Keywords:** Anti-VEGF-A, Angiopoietin 2, Neovascular age-related macular degeneration, Diabetic macular edema, Faricimab, Intravitreal injection, Brazil, Latin America

## Abstract

**Background:**

To investigate real-world use of faricimab for the treatment of neovascular age-related macular degeneration (nAMD) and diabetic macular edema (DME).

**Methods:**

In this first analysis of the BrazilRetNet study group, we performed a multicenter, retrospective chart review of patients who completed the first three intravitreal injections of faricimab until October 2025; patients were followed in 15 retinal centers across Brazil. Collected data included clinical and demographic information, treatment strategy and history; main outcome measures were best-corrected visual acuity (BCVA) converted in ETDRS, macular central subfield thickness (CST) measured on optical coherence tomography (OCT), and adverse events. For comparison among baseline and follow-up variables, Wilcoxon test was employed; p values ≦ 0.05 were considered statistically significant.

**Results:**

A total of 220 patients’ eyes were included. 145 eyes of 127 nAMD patients (56% women, average 78.6 ± 9.4 years-old, 94.5% caucasian) and 75 eyes of 54 DME patients (63% men, average 66.6 ± 10.9 years old, 83.3% caucasian). Prior intravitreal treatment was reported in 123 eyes (84.8%) with nAMD and 60 eyes (80%) with DME, with a mean of 16.8 and 11.7 previous injections, respectively. Baseline mean BCVA was 57.09 (nAMD) and 63.54 (DME). After three injections, mean BCVA improved to 61.06 for nAMD (*p* < 0.001; average gain of 3.97 letters) and 70.27 for DME (*p* < 0.001; average gain of 6.73 ETDRS letters). Baseline mean CST was 320.4 μm (nAMD) and 380.4 μm (DME), decreased to 264.3 μm (nAMD; *p* < 0.001; − 56.1 μm) and 312.7 μm (DME; *p* < 0.001; − 67.7 μm). Differences on BCVA and CST remained statistically significant when subgroups of naive or previously treated eyes were analyzed. All reported adverse events occurred in nAMD patients and included submacular hemorrhage (2 eyes), RPE tear (1 eye) and increased intraocular pressure (1 eye). No intraocular inflammation was reported.

**Conclusions:**

Overall, nAMD and DME patients treated with faricimab in this Brazilian cohort, presented BCVA improvement and a decreased CST after three injections. A low incidence of adverse events was reported, mostly associated to the natural history of the disease. Future analyses will provide further information on real-world farcimab treatment for nAMD and DME in Brazil.

## Background

The identification of vascular endothelial growth factor (VEGF) as a key pro-angiogenic factor involved in vascular permeability and neovascularization represented a major breakthrough in the treatment of retinal vascular diseases, leading to the widespread adoption of anti-VEGF therapies [1]. However, despite anti-VEGF agents becoming the standard of care for age-related macular degeneration (AMD) and diabetic macular edema (DME), treatment typically requires frequent intravitreal injections imposing a substantial burden on patients and caregivers [2]. Moreover, long-term follow-up studies indicate progressive visual decline in a significant proportion of patients, underscoring the need for alternative or complementary therapeutic strategies [3, 4]. 

The relatively short-lasting activity of conventional anti-VEGF compounds translates into the need for frequent retreatments over prolonged periods, with treatment response being highly variable across different studies and patient populations [5, 6]. Real-world evidence from large cohort studies demonstrates that baseline visual acuity in neovascular AMD patients ranges from 50.7 to 53.6 ETDRS letters, and despite anti-VEGF therapy, visual outcomes frequently decline over time, with cumulative losses of approximately 5.2 ETDRS letters by year 4 of treatment [7]. Furthermore, the rate of suboptimal response to anti-VEGF therapy ranges between 30% and 50%, highlighting a significant clinical challenge in managing AMD and DME patients [5, 8]. 

Faricimab is a novel bispecific anti-angiogenic agent that simultaneously inhibits vascular endothelial growth factor A (VEGF-A) and angiopoietin-2 (ANG2). This dual inhibition is particularly relevant given the pathological upregulation of ANG2 in retinal vascular diseases [9]. 

The interaction between ANG2 and the Tie2 receptor activates specific signaling pathways involved in inflammation, angiogenesis, and vascular permeability [10, 11]. Regarding the inflammatory pathway, when the Tie2 receptor is activated by ANG1, ABIN2 (A20-binding inhibitor of NF-κB2) suppresses nuclear factor kappa B (NF-κB) signaling. In contrast, activation of Tie2 by ANG2 leads to the release of NF-κB, which subsequently regulates the transcription of genes associated with inflammation and leukocyte adhesion, including TNF-α, interleukins, ICAM-1, VCAM-1, and E-selectin [12]. 

Concurrently, ANG2 modulates angiogenesis through its interaction with the Tie2 receptor resulting in reduced Tie2 phosphorylation and activation of the FOXO1 transcription factor. Nuclear FOXO1 drives the expression of genes associated with endothelial destabilization and pathological angiogenesis, including ANG2 itself, thereby reinforcing a positive feedback loop that promotes endothelial proliferation, migration, and aberrant vascular sprouting [11]. 

Angiopoietin-1 (ANG1) plays a critical role in maintaining vascular stability by promoting pericyte adhesion to the endothelial cell surface. This interaction strengthens tight junctions and adherens junctions between endothelial cells, thereby reducing vascular leakage and limiting the extravasation of fluid and inflammatory cells. Pericyte–endothelial crosstalk mediated by ANG1–Tie2 signaling is essential for preserving microvascular integrity under both physiological and hypoxic conditions [13]. In contrast, this protective mechanism is disrupted in the presence of ANG2, which induces pericyte detachment from the vessel wall, leading to endothelial destabilization, increased vascular permeability, and structural vascular instability commonly observed in pathological retinal angiogenesis [14–16]. 

Phase 3 pivotal clinical trials are essential for establishing the safety, efficacy, and durability of new therapeutic agents in retinal diseases [17]. In diabetic macular edema, the YOSEMITE and RHINE trials demonstrated that faricimab provided superior anatomical outcomes and greater treatment durability compared with aflibercept 2mg [18]. Regarding retinal fluid control, faricimab achieved a 53% greater reduction in leakage area at week 16, already demonstrating superiority during the head-to-head comparison phase corresponding to the loading dose period [19]. 

Long-term data from the RHONE-X extension study demonstrated that improvements in retinal thickness and fluid control were maintained through the end of the fourth year in patients treated with faricimab. Importantly, patients who switched from aflibercept 2 mg to faricimab during the second year achieved anatomical outcomes comparable to those of patients originally assigned to faricimab by the end of the fourth year. At the conclusion of year four, more than 50% of patients met criteria for treatment intervals longer than 20 weeks, and approximately 77–78% were able to maintain dosing intervals of at least 12 weeks, highlighting the long-term durability of faricimab in DME [20, 21]. 

In neovascular age-related macular degeneration, the pivotal TENAYA and LUCERNE trials similarly demonstrated the superiority of faricimab compared with aflibercept in terms of faster retinal drying during the loading phase and greater treatment durability, while maintaining comparable visual outcomes [22]. Extended follow-up from the AVONELLE-X study showed that visual acuity gains remained stable at the end of four years, with nearly 80% of patients maintaining treatment intervals of 12 weeks or longer, further supporting the sustained efficacy and durability of faricimab in nAMD [23]. 

Although pivotal clinical trials are critical for evaluating the safety and efficacy of new therapies, their strict inclusion and exclusion criteria may limit the applicability of results to routine clinical practice. Consequently, real-world studies have become increasingly important for assessing treatment responses in patient populations that are underrepresented in pivotal trials [24, 25]. Brazil, which has the largest population in Latin America and is one of the most genetically admixed populations worldwide due to the combined genomic heritage of European, African, and Indigenous ancestries, represents a particularly relevant setting for such evaluations [26, 27]. This extensive genetic diversity may influence disease characteristics and therapeutic response, potentially leading to outcomes that differ from those observed in pivotal trials.

For this reason, the first Brazilian real-world research network dedicated to retinal diseases, BrazilRetNet, was established. This network comprises 27 research centers distributed across all regions of Brazil, providing broad geographic and demographic representation. It was specifically designed to generate real-world evidence that complements pivotal clinical trial data and reflects outcomes in a highly diverse, understudied population.

The objective of the present study is to evaluate the real-world response of Brazilian patients with diabetic macular edema and neovascular age-related macular degeneration treated with faricimab, with a particular focus on functional and anatomical outcomes, safety profile and treatment performance in previously treated eyes. By analyzing outcomes from a large multicenter Brazilian cohort, this study aims to expand the current evidence beyond pivotal trial populations and to enhance the understanding of faricimab efficacy, durability, and safety across diverse genetic backgrounds and real-world clinical settings in Latin America.

## Materials and methods

### Study design and patient selection

**Fig. 1:**
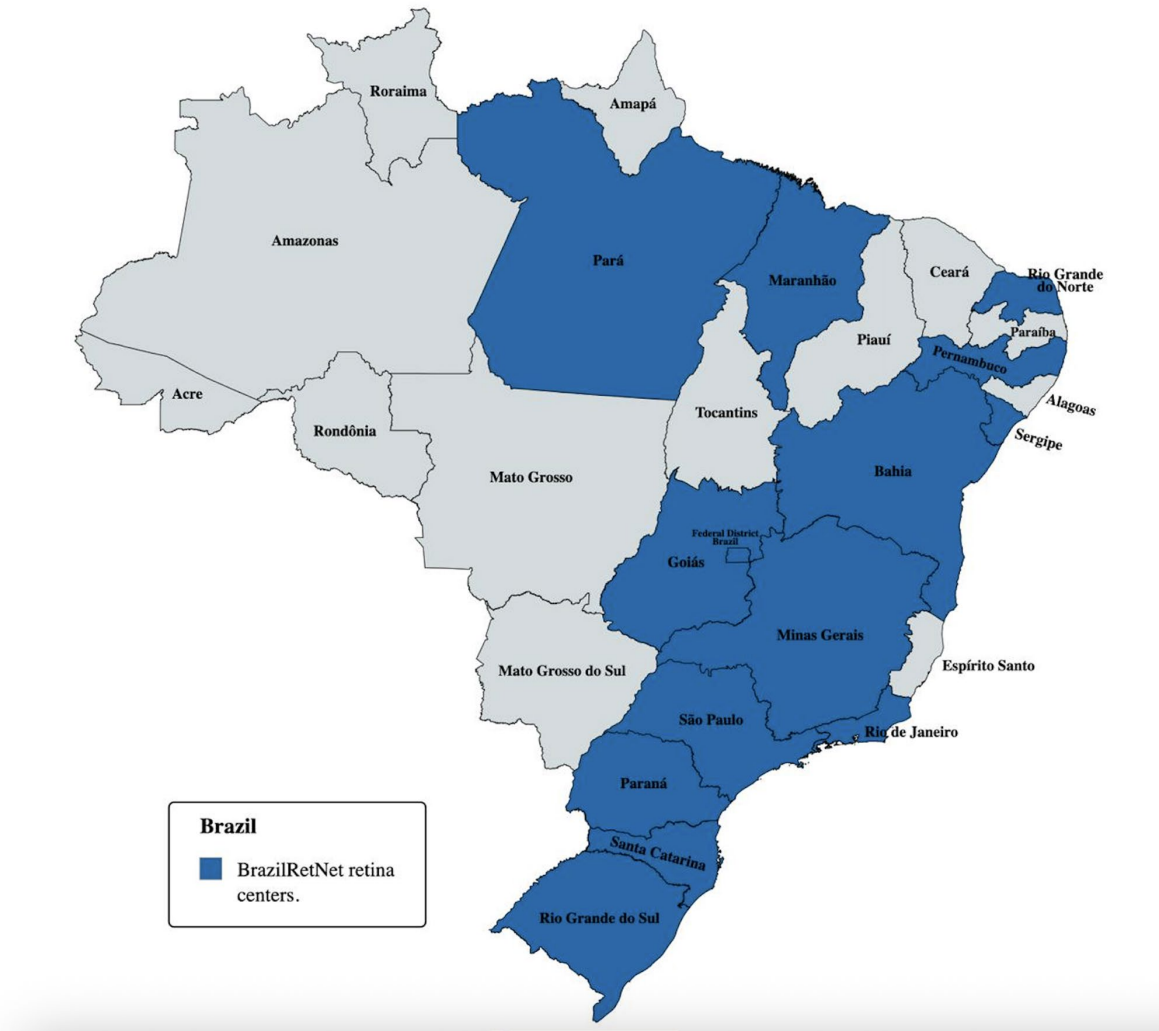
Multicenter distribution of study sites across Brazil. Map of Brazil illustrating the geographic distribution of participating centers. The blue-shaded states indicate the 14 states (15 centers) included in the study, covering an area of approximately 8.5 million km²

This multicenter, retrospective, observational study was conducted by the BrazilRetNet (Brazilian Retinal Research Network), of the 27 participating centers in the network, 15 retinal specialty centers distributed across all geographic regions of Brazil contributed patients to this first report. (Fig. 1). The study protocol was approved by the institutional review boards of the participating centers (CAAE 79630824.1.0000.5370), and the study adhered to the tenets of the Declaration of Helsinki.

Eligible patients were required to have completed three consecutive intravitreal faricimab injections between January 2024 and October 2025, with documented clinical evaluations at each treatment visit. Eyes diagnosed with neovascular age-related macular degeneration or diabetic macular edema, confirmed by multimodal retinal imaging, were included. Exclusion criteria comprised incomplete medical records, presence of concomitant retinal diseases that could confound functional or anatomical outcomes, and insufficient imaging quality precluding reliable measurements.

### Data collection and outcome measures

Data were retrospectively extracted from medical records and included demographic variables (age, sex, ethnicity), clinical diagnosis, treatment history (including prior intravitreal agents and total number of previous injections), and treatment regimen at initiation.

The primary outcome measures were changes in best-corrected visual acuity (BCVA) and central subfield thickness (CST). BCVA was recorded using Snellen charts and converted to Early Treatment Diabetic Retinopathy Study (ETDRS) letter scores using validated method presented by Gregori, Feuer, and Rosenfeld [28]. The conversion of Snellen visual acuity fractions to approximate ETDRS letter scores is used to facilitate statistical analysis of retrospective data. Optical coherence tomography (OCT) imaging was performed using different devices across participating centers, including Nidek RS-330, Heidelberg Spectralis, Zeiss Cirrus platforms (HD, 5000, and 6000), Topcon Triton, and Optovue systems (Solix, Avanti, and RTVue). Although device allocation varied by center, each patient was consistently evaluated using the same OCT device throughout follow-up, minimizing intra-subject variability. While reporting percentage changes in CST could potentially reduce inter-device variability, absolute measurements were maintained to allow comparability with prior clinical trials and real-world studies. CST was assessed by OCT and defined as the mean retinal thickness within the central 1-mm subfield.

Secondary outcomes included the occurrence of ocular adverse events, which were recorded and classified according to type and clinical relevance.

### Treatment protocol

All patients received intravitreal injections of faricimab (6.0 mg/0.05 mL) administered under sterile conditions in accordance with local institutional protocols. Treatment strategies, including initiation of therapy or switching from previous anti–vascular endothelial growth factor (anti-VEGF) agents, were determined by the treating physician based on individual clinical judgment. Reasons for switching included inadequate anatomical or functional response to prior therapies, treatment burden, or physician preference.

### Statistical analysis

Continuous variables were expressed as mean ± standard deviation. Comparisons between baseline and post-treatment outcomes were performed using the Wilcoxon signed-rank test due to non-normal data distribution. Prespecified subgroup analyses were conducted according to treatment status (treatment-naïve versus previously treated eyes). Statistical significance was defined as a two-sided p value < 0.05. All analyses were performed using appropriate statistical software.

## Results

### Patient demographics and baseline characteristics

A total of 220 eyes from 181 patients were included, comprising 145 eyes of 127 patients with nAMD and 75 eyes of 54 patients with DME.

In the nAMD cohort, 56% of patients were female, with a mean age of 78.6 ± 9.4 years, and 94.5% were Caucasian. In the DME cohort, 63% were male, the mean age was 66.6 ± 10.9 years, and 83.3% were Caucasian.

Prior intravitreal treatment was reported in 123 eyes (84.8%) with nAMD and 60 eyes (80%) with DME, with a mean of 16.8 and 11.7 previous injections, respectively. (Table 1)

**Table 1:** Patient Demographics and Baseline Characteristics

Characteristic	nAMD	DME
Number of eyes	145	75
Number of patients	127	54
Female, n (%)	71 (56.0%)	20 (37.0%)
Male, n (%)	56 (44.0%)	34 (63.0%)
Mean age, years (SD)	78.6 ± 9.4	66.6 ± 10.9
Caucasian, n (%)	120 (94.5%)	45 (83.3%)
Treatment-naïve	22 eyes (15.1%)	15 eyes (20%)
Previously treated	123 eyes (84.8%)	60 eyes (80%)
Mean previous injections	16,8	11,7

### Visual acuity outcomes

In the overall nAMD cohort, mean baseline BCVA was 57.09 ETDRS letters. After three injections, mean BCVA improved to 61.06 letters, corresponding to a mean gain of 3.97 letters, which was statistically significant (*p* < 0.001). In DME eyes, mean baseline BCVA was 63.54 ETDRS letters, increasing to 70.27 letters after three injections, with a mean gain of 6.73 letters (*p* < 0.001). (Table 2)

**Table 2:** Combined Functional and Anatomical Outcomes (Overall)

Outcome	nAMD (*n* = 145)	DME (*n* = 75)
Baseline BCVA (letters)	57,09	63,54
BCVA after 3 injections	61,06	70,27
Mean BCVA gain	3,97	6,73
Baseline CST (µm)	320,4	380,4
CST after 3 injections (µm)	264,3	312,7
Mean CST reduction (µm)	-56,1	-67,7
Overall p-value	< 0.001	< 0.001

### Anatomical outcomes

Mean baseline CST in nAMD eyes was 320.4 μm, decreasing to 264.3 μm after three injections, corresponding to a mean reduction of 56.1 μm (*p* < 0.001). In DME eyes, mean CST decreased from 380.4 μm at baseline to 312.7 μm after treatment, representing a mean reduction of 67.7 μm (*p* < 0.001). (Table 2)

### Subgroup analysis: diabetic macular edema

Among treatment-naïve DME eyes (*n* = 15), mean BCVA improved from 63.75 to 72.18 ETDRS letters, with a mean gain of 8.43 letters (Wilcoxon test, *p* = 0.025). Mean CST decreased from 343.9 μm to 281.8 μm, corresponding to a reduction of 62.1 μm (*p* = 0.02).

In previously treated DME eyes (switch group, *n* = 60), mean BCVA increased from 63.49 to 69.79 ETDRS letters, resulting in a mean gain of 6.3 letters (*p* < 0.001). Mean CST decreased from 389.6 μm to 320.4 μm, with a mean reduction of 69.2 μm (*p* < 0.001). (Table 3)

**Table 3:** Subgroup Analysis (Naïve vs. Switch)

Outcome	DME Naïve (*n* = 15)	DME Switch (*n* = 60)	nAMD Naïve (*n* = 22)	nAMD Switch (*n* = 123)
Baseline BCVA	63,75	63,49	54,11	57,38
BCVA after 3 injections	72,18	69,79	63,73	60,58
Mean letter gain	8,43	6,3	9,62	3,2
p-value	0.025	< 0.001	0.016	< 0.001
Baseline CST (µm)	343,9	389,6	404,7	305,3
Post-treatment CST (µm)	281,8	320,4	258,7	265,3
Mean CST reduction (µm)	-62,1	-69,2	-146	-40
p-value (CST)	0.02	< 0.001	< 0.001	< 0.001

### Subgroup analysis: neovascular age-related macular degeneration

In the nAMD switch subgroup (*n* = 123), mean BCVA improved from 57.38 to 60.58 ETDRS letters, corresponding to a mean gain of 3.2 letters (*p* < 0.001). Mean CST decreased from 305.3 μm to 265.3 μm, representing a mean reduction of 40.0 μm (*p* < 0.001).

In treatment-naïve nAMD eyes (*n* = 22), mean BCVA increased from 54.11 to 63.73 ETDRS letters, with a mean gain of 9.62 letters (*p* = 0.016). Mean CST showed a pronounced reduction from 404.7 μm at baseline to 258.7 μm post-treatment, corresponding to a mean decrease of 146.0 μm (*p* < 0.001). (Table 3)

### Treatment regimens

At treatment initiation, the most frequently planned regimen for nAMD was treat-and-extend (T&E) accounting for 81 eyes (55.9%). A loading-dose-only strategy was planned in 25 eyes (17.2%), while PRN regimens were used in 35 eyes (24.1%). Other individualized regimens accounted for 4 eyes (2.8%).

In the DME cohort, T&E regimens planned in 32 eyes (42.7%). A loading-dose-only approach was used in 26 eyes (34.7%), PRN in 15 eyes (20.0%), and other regimens in 2 eyes (2.6%).

### Safety

All adverse events were observed in the nAMD cohort and included submacular hemorrhage in two eyes, retinal pigment epithelium tear in one eye, and increased intraocular pressure in one eye. No treatment-related adverse events were reported in the DME group.

## Discussion

Faricimab was approved for clinical use in Brazil by the Brazilian Health Regulatory Agency (Agência Nacional de Vigilância Sanitária—ANVISA) in late 2023. Following its approval, the use of faricimab has progressively increased, driven by the favorable outcomes demonstrated in pivotal clinical trials. These trials showed superiority in outcomes related to retinal fluid resolution, including a greater proportion of patients achieving fluid-free status, faster time to fluid clearance, and the ability to extend treatment intervals [18, 22]. Collectively, these characteristics have been associated with improved visual outcomes, better patient adherence, and a reduction in treatment burden.

However, current therapeutic decision-making has largely been guided by data derived from pivotal trials and real-world studies conducted predominantly in North America and Europe. These populations differ substantially from Brazilian and Latin American populations in terms of genetic background, socioeconomic conditions, access to healthcare, and disease characteristics. As a result, treatment responses observed in these regions may not fully reflect outcomes in Brazilian patients.

To address this gap, the BrazilRetNet was established as a collaborative group of retinal specialists dedicated to conducting real-world research focused on the Brazilian population. The primary goal of this initiative is to generate population-specific evidence to support clinical decision-making and reduce the risk of unexpected outcomes related to both efficacy and safety when new therapies are introduced. The present study represents the first investigation conducted by the BrazilRetNet consortium and evaluates real-world outcomes of faricimab therapy in patients with DME and nAMD, contributing important data from a highly diverse and genetically admixed population that has been underrepresented in pivotal clinical trials.

### Visual acuity and anatomical outcomes in neovascular age-related macular degeneration

In our nAMD cohort, visual acuity outcomes demonstrated an early functional benefit, with a mean gain of approximately + 4 ETDRS letters after the first faricimab injection. This improvement was statistically significant and numerically greater than that reported in the TRUCKEE real-world study, which observed a mean gain of + 1.1 letters after the initial injection. In TRUCKEE, visual acuity gains were + 0.2 letters in previously treated (switch) eyes and + 4.9 letters in treatment-naïve eyes, highlighting a more pronounced early response in naïve patients [29]. 

After three injections, visual acuity gains in our study remained stable, with a mean improvement of + 3.97 letters. Subgroup analyses demonstrated a gain of + 3.2 letters in previously treated eyes and a more pronounced gain of + 9.62 letters in treatment-naïve eyes. This stabilization of visual acuity following an early functional response suggests that the initial benefit observed after the first injection is maintained through the follow-up. This pattern has been consistently described in pivotal clinical trials and corroborated by real-world evidence. Notably, the TRUCKEE study reported a comparable overall gain of + 3.4 letters after three injections, with treatment-naïve eyes achieving gains of up to + 8.1 letters [29]. 

The maintenance of visual acuity improvement from the first injection through completion of the three-injection contrasts with the slower or more gradual gains reported with some other anti-VEGF agents. This finding supports the hypothesis that faricimab’s prolonged intraocular durability and its enhanced inhibition of vascular permeability through angiopoietin-2 blockade may contribute to sustained functional benefits. Importantly, early response to anti-VEGF therapy has been proposed as a potential predictor of long-term outcomes, underscoring the clinical relevance of the rapid functional improvement observed in our cohort [30]. 

From an anatomical perspective, our study demonstrated a mean overall reduction in central macular thickness of − 56.1 μm in nAMD eyes. When stratified by treatment status, previously treated eyes showed a mean reduction of − 40 μm, whereas treatment-naïve eyes exhibited a substantially greater decrease of − 146 μm. These findings indicate that faricimab achieves rapid and clinically meaningful anatomical improvement from the early stages of treatment.

These anatomical outcomes compare favorably with the TRUCKEE real-world study, which reported an overall CST reduction of − 43 μm, including reductions of − 38.1 μm in switch eyes and − 80.1 μm in treatment-naïve eyes [29]. The numerical similarity and, in some subgroups, greater magnitude of anatomical response observed in our cohort support the external validity of our findings and suggest that the anatomical benefits of faricimab are reproducible across different clinical settings and healthcare systems.

Consistent results have also been reported in the FARETINA-AMD real-world cohort, in which mean CST in treatment-naïve eyes decreased from approximately 315 μm at baseline to 265 μm after four injections, while previously treated eyes showed a reduction from approximately 296 μm to 273 μm over the same period [31]. The convergence of anatomical outcomes across our study and large registry-based real-world analyses reinforces the robustness and consistency of faricimab-associated anatomical improvements in routine clinical practice.

Taken together, these findings indicate that faricimab induces a clinically meaningful and early anatomical response, particularly in treatment-naïve eyes, while also providing relevant anatomical benefit in previously treated cases. The alignment of our results with both real-world evidence and pivotal clinical trials supports the use of faricimab as an effective therapeutic option both as first-line therapy and as a switch strategy in nAMD.

### Visual acuity and anatomical outcomes in diabetic macular edema

In the DME cohort, faricimab therapy resulted in a mean visual acuity gain of + 6.73 ETDRS letters after three injections. Subgroup analyses demonstrated gains of + 6.3 letters in previously treated eyes and + 8.43 letters in treatment-naïve eyes, indicating a robust functional response even among patients with prior exposure to anti-VEGF therapy.

These findings are consistent with recently presented data from the RHONE-X study, an extension of the RHINE trial, which demonstrated that patients who switched from aflibercept to faricimab were able to achieve visual acuity outcomes comparable to those of patients receiving continuous faricimab therapy by the end of the fourth year. Notably, prior to switching, a smaller proportion of patients treated with aflibercept achieved CST < 325 μm (90%) compared with those receiving faricimab (97–98%). After transitioning to faricimab, similar proportions of patients reached CST < 325 μm (99% in the original faricimab arm and 98% in the prior aflibercept arm), a finding that may support the potential benefit of switching therapy in selected patients [20]. 

Real-world evidence from the FARWIDE-DMO study reported a mean visual acuity gain of + 4.7 letters at 12 months in treatment-naïve eyes, whereas previously treated eyes exhibited relatively stable vision with a mean change of − 1.2 letters. When stratified by baseline visual acuity (56–69 letters), treatment-naïve eyes achieved a gain of + 6.2 letters, comparable to the improvement observed in our treatment-naïve cohort. In contrast, switch patients in FARWIDE-DMO demonstrated a more modest gain of + 1.8 letters, which differs from the more pronounced early improvement observed in our switch cohort [32]. 

Preliminary findings from the TAHOE real-world study further support the functional efficacy of faricimab in DME, reporting a mean visual acuity gain of + 2.16 letters after three injections, although this improvement was lower than that observed in our cohort [33]. 

Regarding anatomical outcomes, our DME patients demonstrated a mean CMT reduction of − 67.7 μm, with reductions of − 69.2 μm in switch eyes and − 62.1 μm in treatment-naïve eyes. These reductions exceed those reported in other real-world studies, including TAHOE and FARETINA-DME, which reported mean CMT reductions ranging from approximately − 45 to − 51 μm [33, 34]. The greater magnitude of anatomical improvement observed in our cohort suggests a pronounced early response following faricimab initiation, which may be related to baseline disease severity, treatment timing, or population-specific characteristics.

Importantly, the Brazilian population represents one of the most genetically admixed populations worldwide, with contributions from European, African, and Indigenous ancestries [26, 27]. This population heterogeneity may influence treatment response and is not fully represented in pivotal clinical trials or prior real-world studies. The robust functional and anatomical outcomes observed in our cohort therefore provide valuable evidence supporting the effectiveness of faricimab in a diverse and underrepresented population.

### Safety profile

Regarding safety, faricimab demonstrated a profile consistent with previously published real-world evidence. The incidence and nature of adverse events observed in our cohort aligned with findings from large observational studies, including FARWIDE, FARETINA, TRUCKEE, and VOYAGER. No cases of retinal vasculitis were observed, an important finding given concerns related to intraocular inflammation with other recently introduced anti-VEGF agents.

One case of retinal pigment epithelium tear was observed in the nAMD cohort. RPE tears are a recognized complication of anti-angiogenic therapy and are more closely associated with disease-related factors—such as large fibrovascular pigment epithelial detachments and rapid contraction of the neovascular complex—than with a specific pharmacologic agent. Two cases of submacular hemorrhage were identified, a complication historically reported with all anti-VEGF therapies. Although a recent retrospective analysis suggested a lower overall incidence of submacular hemorrhage with faricimab, hemorrhagic events tended to occur earlier in the treatment course, a finding consistent with the short-term focus of the present analysis. A single case of increased intraocular pressure was observed, a well-recognized and typically transient complication of intravitreal injection, with an incidence consistent with previously reported rates.

### Treatment durability and clinical considerations

Treatment regimens were heterogeneous, reflecting the real-world and retrospective nature of this study. Therapeutic decisions, including treatment indication and regimen selection (loading dose, treat-and-extend, or pro re nata), were determined at the discretion of the attending physician based on individual patient characteristics and disease status at the time of presentation. As the study was not yet conceived during the treatment period, no standardized protocol or external influence guided the use of faricimab. Consequently, patients were included at different stages of their treatment course, contributing to variability in exposure and follow-up. While this heterogeneity may have influenced the observed outcomes, it also enhances the external validity of the findings by capturing routine clinical practice. Treat-and-extend was the most frequently planned regimen in both nAMD and DME cohorts. In the treatment-naïve patients, the achievement of significant functional and anatomical improvements during the initial three-injection provides a strong rationale for extended dosing intervals. Pivotal trial data have demonstrated that a substantial proportion of patients can maintain treatment intervals of 12 weeks or longer, supporting the durability of faricimab’s therapeutic effect and reinforcing the role of treat-and-extend strategies in routine clinical care [18, 22]. 

### Study limitations and strengths

This study has limitations inherent to its retrospective design and focus on early outcomes following three injections. The multicenter nature of the data collection involved the use of optical coherence tomography (OCT) devices from different manufacturers, and variability in segmentation algorithms and acquisition protocols across these devices may impact the accuracy and comparability of absolute central subfield thickness (CST) measurements, constituting an inherent limitation of this study. Furthermore, intravitreal injections were performed by multiple physicians, potentially leading to slight variations in clinical technique. Longer-term follow-up will be required to assess durability, extended dosing intervals, and long-term safety. Additionally, the relatively small number of treatment-naïve eyes limits subgroup generalizability.

The statistical analysis was primarily based on within-subject comparisons between baseline and post-treatment outcomes using the Wilcoxon signed-rank test. While this approach is appropriate for assessing early changes in a real-world setting, it does not account for potential confounding factors such as inter-center variability, differences in treatment regimens, or baseline characteristics between treatment-naïve and previously treated eyes. Additionally, no multivariable modeling was performed to adjust for these variables. Therefore, the results should be interpreted with caution. Future studies with larger cohorts and longer follow-up are warranted to enable more comprehensive statistical analyses and to better control for potential confounders.

Nevertheless, the study’s strengths include its multicenter design, broad geographic representation, and inclusion of a highly diverse population. The consistency of our findings with large registry-based real-world studies enhances confidence in the robustness and external validity of the results.

## Conclusions

This multicenter Brazilian real-world study demonstrates that faricimab provides significant functional and anatomical benefits in both neovascular age-related macular degeneration and diabetic macular edema, including in patients with extensive prior anti-VEGF exposure. Given the distinct pathophysiological mechanisms of these conditions, outcomes should be interpreted within each disease context, although consistent early benefits were observed across both cohorts.

The favorable safety profile and robust early treatment response support faricimab’s use as both a first-line and switch therapy in nAMD and DME. These findings complement pivotal clinical trial data and provide important real-world evidence supporting the applicability of faricimab across diverse populations and clinical settings in Latin America.

## Data Availability

The datasets used and/or analysed during the current study are available from the corresponding author on reasonable request.

## Abbreviations

namd: Neovascular age-related macular degeneration
DME: Diabetic macular edema
BCVA: Best-corrected visual acuity
CST: Macular central subfield thickness
OCT: Optical coherence tomography
VEGF: Vascular endothelial growth factor
VEGF-A: Vascular endothelial growth factor A
ANG2: Angiopoietin-2
ABIN2: A20-binding inhibitor of NF-κB2
NF-κB: Nuclear factor kappa B
ANG1: Angiopoietin-1
BrazilRetNet: Brazilian Retinal Network
ETDRS: Early Treatment Diabetic Retinopathy Study
Anti-VEGF: Anti–vascular endothelial growth factor
